# Molecular Detection of Tick-Borne Parasitic Pathogens in Ticks Collected from Animal Hosts in Chuy Province, Kyrgyzstan

**DOI:** 10.3390/pathogens15090940

**Published:** 2026-09-04

**Authors:** Hyun Jung Kim, Ji Ye Seo, Jin Seo Park, Jung-Won Ju, Bekbolsun Aknazarov, Nurzina Atabekova, Sang-Eun Lee, Hee-Il Lee

**Affiliations:** 1 Division of Vectors and Parasitic Diseases, Korea Disease Control and Prevention Agency (KDCA), Cheongju 28159, Republic of Korea; 2 Department of Medical Science and Infectious Biology, College of Medicine, Chungnam National University, Daejeon 35015, Republic of Korea; 3 Faculty of Veterinary Medicine and Biotechnology, Kyrgyz National Agrarian University Named After Konstantin Ivanovich Skryabin, Bishkek 720005, Kyrgyzstan

**Keywords:** Kyrgyzstan, tick-borne parasites, *Babesia*, *Theileria*

## Abstract

Tick-borne parasitic diseases pose important threats to veterinary and public health, particularly in countries with livestock-dependent economies such as Kyrgyzstan. However, molecular epidemiological data on tick-borne pathogens in Kyrgyzstan is limited. This study investigated the prevalence, genetic diversity, and host–tick–pathogen associations in Chuy Province, Kyrgyzstan. A total of 494 ticks belonging to 6 genera and 10 species were collected from various animal hosts in eight survey areas in central-northern Chuy Province and analyzed using polymerase chain reaction, Sanger sequencing, and phylogenetic analysis. Of the 494 ticks, 35 (7.1%) were infected, including 15 (3.0%) with *Babesia* species and 20 (4.0%) with *Theileria* species. The highest positive rates were observed in *Rhipicephalus annulatus* (13.8%) and *Haemaphysalis punctata* (12.4%). Molecular analysis identified 4 *Babesia* species (*B. caballi*, *B. major*, *B. bigemina*, *B. occultans*) and 2 *Theileria* species (*T. orientalis*, *T. ovis*). Ticks collected from horses had the highest infection rates and greatest diversity of pathogens. *T. orientalis* was the most common pathogen. *T. ovis* was exclusively found in ticks collected from sheep. Phylogenetic analysis confirmed that the isolates were closely related to strains circulating in neighboring Central Asian and Eurasian countries. The findings provide foundational data for developing region-specific vector control measures.

## 1. Introduction

Tick-borne diseases are a threat to both public health and veterinary care worldwide [1,2]. Recent climate change, characterized by rising temperatures and humidity, has led to ecological shifts in tick vectors, including expansion of their geographical distribution and extension of their active periods [3,4]. Consequently, outbreaks of tick-borne diseases are increasingly being reported in high-latitude and high-altitude areas where they have not occurred historically [5,6,7]. Therefore, implementing a continuous systematic surveillance system to proactively identify and manage the expansion of tick habitats and pathogen transmission pathways is an urgent global challenge.

Among the various tick-borne pathogens, parasitic pathogens such as *Babesia* and *Theileria* are critical not only because of their direct impact on the livestock economy but also threat to public health [8,9,10]. These protozoan parasites are transmitted to cattle, sheep, and goats by blood-feeding ticks, causing severe anemia, high fever, and loss of appetite, which can be fatal and results in substantial economic losses for livestock farms. Furthermore, these parasitic pathogens can be transmitted to humans by tick bites. Specifically, some *Babesia* species cause human babesiosis, an acute febrile zoonotic disease that can be fatal in immunocompromised individuals and those with underlying conditions [11,12,13]. Understanding the infection mechanisms and distribution of parasitic pathogens within ticks is therefore critical not only for establishing animal disease control systems but also for building a One Health-based integrated public health surveillance system to capable of preemptively breaking the transmission cycle to humans [14].

Kyrgyzstan, located in Central Asia, is composed mostly of mountainous terrain and vast grasslands, and the national economy depends on grazing-based livestock farming [15]. These geographical and environmental characteristics provide favorable conditions for various tick species, including hard ticks, to persist and encounter livestock. However, epidemiological research on tick-borne parasites in Kyrgyzstan is limited. Previous studies of tick-borne pathogens that infect livestock have mainly focused on direct detection of parasitic pathogens in animal blood or have restricted pathogen identification to the genus level [16,17,18,19,20]. Consequently, these studies have lacked the species-level molecular data necessary to accurately characterize the epidemiology of tick-borne parasitic diseases in this region.

To address this gap, we investigated the prevalence of *Babesia* and *Theileria* species in livestock-associated ticks in central-northern Chuy Province, characterized the infection patterns according to tick species and host animal, and examined the phylogenetic relationships of circulating *Babesia* and *Theileria* strains with those reported in neighboring Central Asian and Eurasian countries. Species-level molecular identification of *Babesia* and *Theileria* in ticks collected from livestock and other animals in Chuy Province was conducted using polymerase chain reaction (PCR)-based screening, Sanger sequencing, and phylogenetic analysis to characterize host–tick–pathogen association. The results of this study are intended to provide baseline molecular epidemiology data needed to develop vector control and animal disease prevention strategies tailored to Kyrgyzstan and the wider Central Asian region.

## 2. Materials and Methods

### 2.1. Tick Collection and Study Area

**Figure 1:**
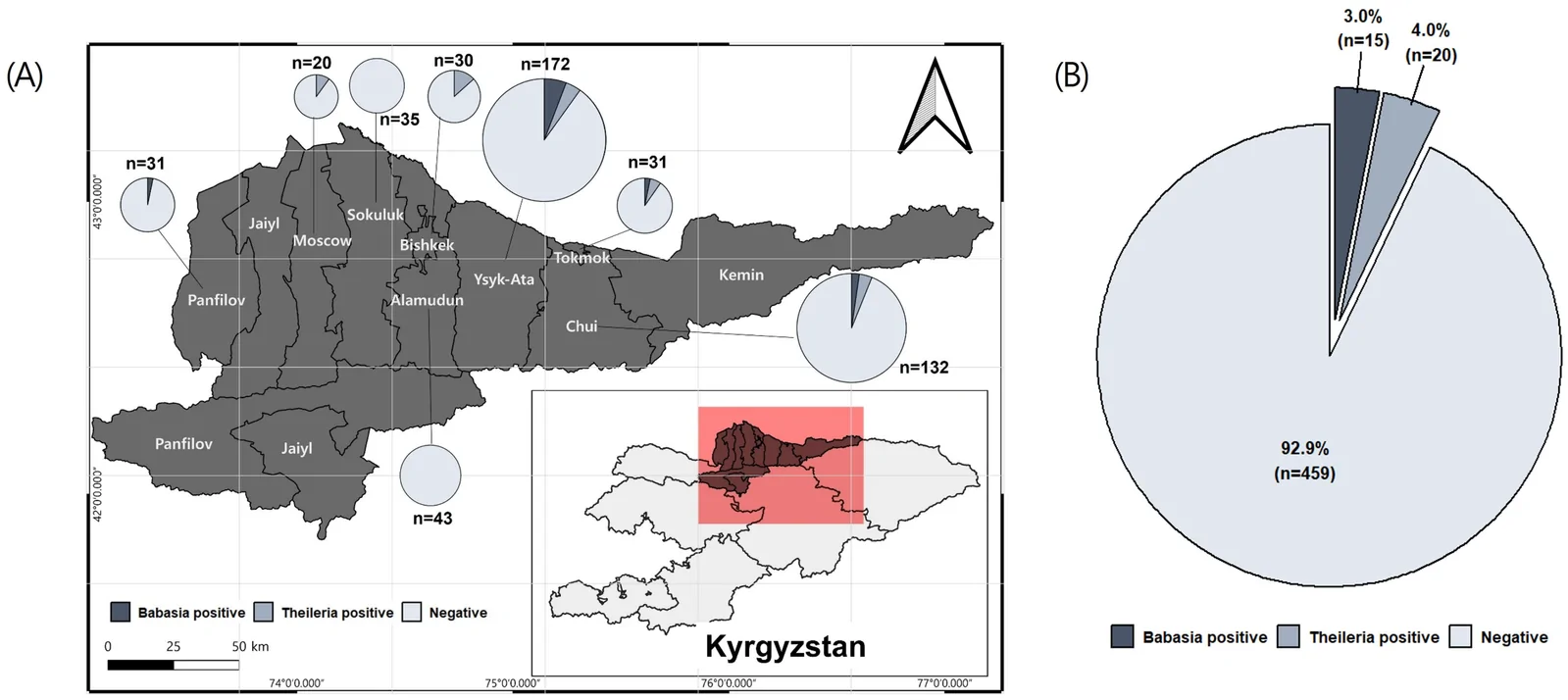
Location of tick sample collection and detection of tick-borne parasites in Chuy Province, Kyrgyzstan. (**A**) Map showing the number of ticks collected and tick-borne parasites carried by the ticks by area. (**B**) Pie chart showing the number and percentage of collected ticks with tick-borne parasites. The Kyrgyzstan map was created in QGIS. Administrative boundaries were derived from the United Nations Office for the Coordination of Humanitarian Affairs (OCHA) shapefiles. Panel (**A**) depicts the same tick collection sites and sampling areas as (**A**) in Kim et al. [21], as both studies analyzed ticks collected from the same area.

This study used 494 ticks collected from animal hosts in a previous study on tick-borne bacterial pathogens by Kim et al. [21]. The ticks were collected from cats, cattle, chickens, dogs, horses, and sheep in six rural areas (Alamudun, Chui, Ysyk-Ata, Moscow, Sokuluk, and Panfilov) and two urban areas (Bishkek and Tokmok) in central-northern Chuy Province in 2021 (Figure 1A). The developmental stage (adult, nymph, and larva) and sex information of all collected ticks were not recorded. Each tick was processed individually for DNA extraction and PCR.

### 2.2. DNA Extraction and Molecular Detection of Tick-Borne Parasites

Genomic DNA was extracted from each tick using the Clear-STM Quick DNA extraction kit (InvirusTech, Gwangju, Republic of Korea). Briefly, ticks were homogenized using a Precellys 24 tissue homogenizer (30 Hz for 2 min; Bertin Instruments, Montigny-le-Bretonux, France) with 600 µL of lysis solution and 2.8 mm ceramic (zirconium oxide) beads. Following the manufacturer’s instructions, the tick lysate was centrifuged at 12,000× *g* for 10 min, and the supernatant was used for DNA isolation. The isolated DNA was eluted in 50 µL of elution buffer and stored at −80 °C until further analysis. Tick-borne parasites were detected by conventional PCR using the AccuPower *Babesia* & *Theileria* PCR kit (Bioneer, Daejeon, Republic of Korea), dried-format HotStart PCR premix targeting the *Babesia* and *Theileria* 18S ribosomal RNA gene to obtain a 561 bp fragment according to the manufacturer’s instructions.

Briefly, amplification was performed in a 20 μL reaction volume containing 3 μL of genomic DNA and 17 μL of distilled water. Thermal cycling conditions, per the manufacturer’s protocol, consisted of an initial denaturation at 95 °C for 5 min, followed by 40 cycles of denaturation at 95 °C for 20 s and annealing/extension at 59 °C for 50 s, with a final extension at 72 °C for 5 min. Nuclease-free water and the kit-supplied positive control template were included in each run as the negative and positive controls, respectively. PCR was performed using a C1000 Touch Thermal Cycler (Bio-Rad Laboratories, Hercules, CA, USA), and the amplified products were analyzed by electrophoresis using an automated QIAxcel system (Qiagen, Germantown, MD, USA).

### 2.3. Nucleotide Sequencing and Phylogenetic Analysis

PCR products from pathogen-positive samples were purified and sent to a commercial sequencing service (Bionics, Daejeon, Republic of Korea). Nucleotide sequence homology searches were performed using the National Center for Biotechnology Information (NCBI) BLAST network service (version 2.17.0, NCBI, Bethesda, MD, USA). Multiple sequence alignments were performed using the CLUSTAL Omega (version 1.2.1), yielding trimmed alignments of 617 bp for the *Babesia* dataset and 755 bp for the *Theileria* dataset. Phylogenetic trees were constructed with MEGA 11.0 software using the maximum likelihood method, with *Trypanosoma evansi* (AY904050) used as the outgroup. The best-fit nucleotide substitution model was selected based on the lowest Bayesian information criterion (BIC) value.

### 2.4. Data Analysis

A descriptive analysis, including pie charts and alluvial plots, was conducted using R version 3.4.3 (The R Foundation for Statistical Computing, Vienna, Austria). Alluvial plots were constructed in ggplot2 (version 3.3.3) using the ggalluvial package (version 0.12.3).

## 3. Results

### 3.1. Prevalence of Tick-Borne Parasites by Area

As reported previously [21], 494 ticks were collected from animals in the eight survey areas in central-northern Chuy Province. The number of ticks collected in each survey area ranged from 20 to 172, with the largest number of samples being collected from the Ysyk-Ata (*n* = 172), Chui (*n* = 132), and Alamudun (*n* = 43) areas (Figure 1A). Of the 494 ticks, 15 (3.0%) were positive for *Babesia* species, and 20 (4.0%) were positive for *Theileria* species (Figure 1B). *Babesia* was detected in 4 survey areas (Panfilov, Ysyk-Ata, Tokmok, and Chui), and *Theileria* was detected in 5 survey areas (Moscow, Bishkek, Ysyk-Ata, Tokmok, and Chui). No tick-borne parasitic pathogens were detected in ticks collected in the Sokuluk or Alamudun areas.

### 3.2. Detection of Tick-Borne Parasites by Tick Species

Sanger sequencing identified that the 494 ticks belonged to 10 tick species from 6 genera (*Argas*, *Haemaphysalis*, *Dermacentor*, *Rhipicephalus* and *Hyalomma*) (Table 1). The overall prevalence of tick-borne parasite infection in the ticks was 7.1% (*n* = 35). The prevalence of parasite infection was highest in *R. annulatus* (8/58, 13.8%), followed by *H. punctata* (11/89, 12.4%), *D. marginatus* (4/38, 10.5%), and *H. marginatum* (2/19, 10.5%).

Four *Babesia* species were detected among 15 ticks testing positive for *Babesia*. *B. caballi* and *B. major* were detected primarily in *D. marginatus*, *Dermacentor* spp., and *H. punctata.* The prevalence of *Babesia* was highest in *D. marginatus* (4/38, 10.5%, comprising 3 *B. caballi* and 1 *B. major*), followed by *H. punctata* (5/89, 5.6%). Two cases of *B. bigemina* infection were detected in *R. annulatus* ticks collected from horses, and a single case of *B. occultans* infection was identified in a *Hyalomma scupense* tick collected from a chicken.

Two *Theileria* species were detected among 20 positive ticks belonging to multiple tick genera. *T. orientalis* was the most widely distributed species, detected in *A. persicus*, *Dermacentor* spp., *H. punctata*, and *R. annulatus*, with the highest absolute number of ticks infected with *T. orientalis* being *R. annulatus* (6/58, 10.3%) and *H. punctata* (6/89, 6.7%). *T. ovis* was detected exclusively in *H. marginatum* (2/10) and *R. turanicus* (4/23) collected from sheep. None of the ticks were coinfected with more than one parasite species.

### 3.3. Tick–Host–Pathogen Association

**Figure 2:**
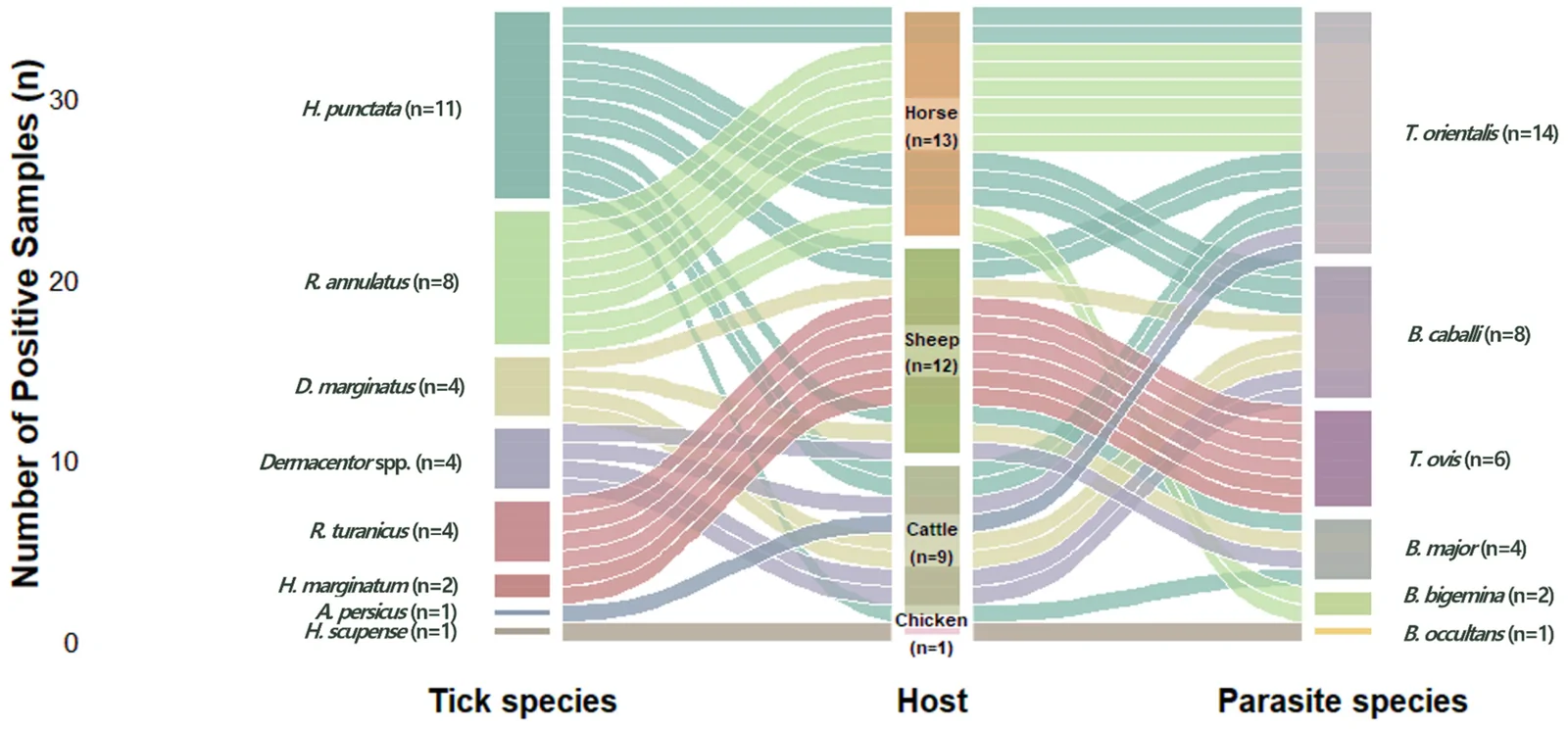
Alluvial plot showing tick–host–pathogen associations. The columns from left to right represent the collected tick species, the corresponding host animal from which the ticks were recovered, and the *Babesia* and *Theileria* species detected in the ticks.

Alluvial analysis of the tick–host–pathogen network identified the transmission pathways linking tick vectors, animal hosts, and parasite species (Figure 2). Among the 35 positive ticks, the highest number of positive ticks (*n* = 13) were collected from horses (13/71, 18.3%). Ticks collected from horses harbored multiple species of pathogens, including *T. orientalis*, *B. caballi*, and *B. bigemina*, which were detected primarily in *R. annulatus* and *H. punctata*. The second most frequent source of positive ticks (*n* = 12) were collected from sheep (12/161, 7.5%). *T. ovis* was detected only in *R. turanicus* and *H. marginatum* collected from sheep, whereas *B. caballi* and *B. major* were detected in *H. punctata*, *D. marginatus*, and *Dermacentor* spp. collected from sheep. Nine positive ticks were collected from cattle (9/122, 7.4%). Ticks collected from cattle were positive mainly for *T. orientalis*, *B. caballi*, and *B. major*, which were detected primarily in *D. marginatus*, *Dermacentor* spp., and *H. punctata*. A single chicken host was positive for *B. occultans* associated with *H. scupense*.

### 3.4. Phylogenetic Analysis of Tick-Borne Parasitic Pathogens

Among the 15 *Babesia*-positive and 20 *Theileria*-positive samples, 6 representative *Babesia* sequences and 2 representative *Theileria* sequences were selected after removing duplicate sequences.

**Figure 3:**
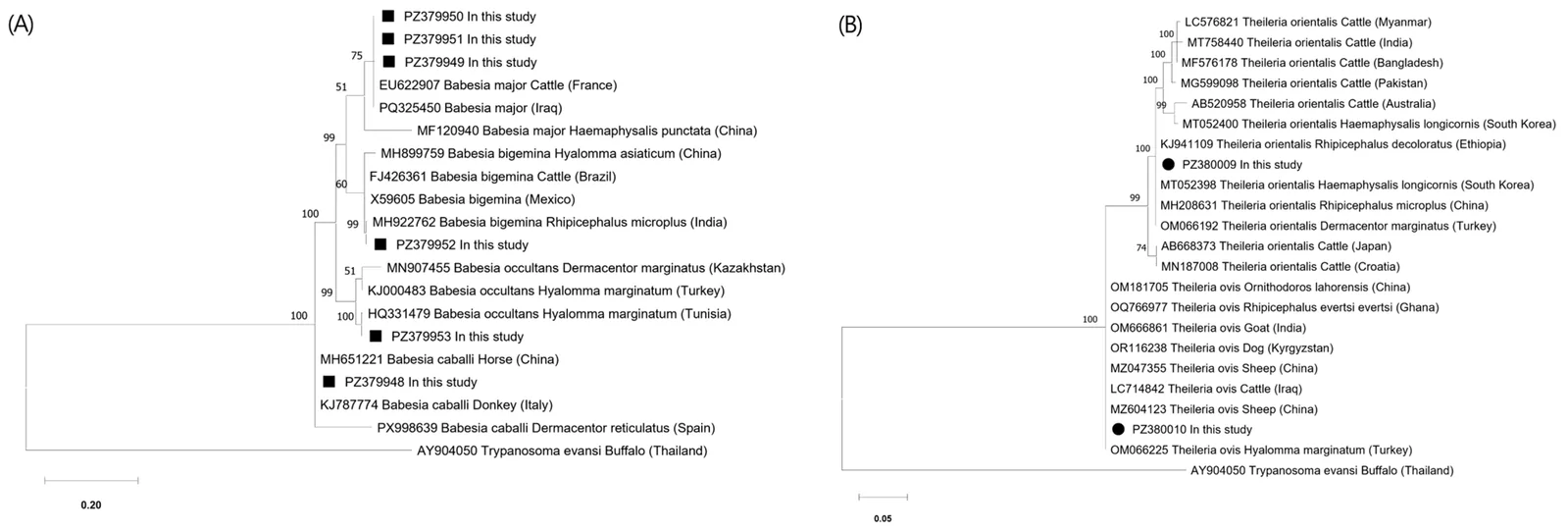
Phylogenetic analysis of tick-borne parasites based on partial nucleotide sequences of (**A**) *Babesia* and (**B**) *Theileria* 18S rRNA. The solid square (■) and circle (●) represent the *Babesia* and *Theileria* sequences of this study, respectively. The phylogenetic trees were constructed using the maximum likelihood method and the Kimura 2-parameter model. Rate variation among sites was modeled using a gamma distribution with a proportion of invariant sites (G + I) in (**A**), and a proportion of invariant sites (I) in (**B**). The number of branches indicate bootstrap percentages based on 1000 replications.

For *Babesia major*, sequences obtained in this study (PZ379949, PZ379950, and PZ379951) revealed a 99.83–100% identity with *B. major* isolated from cattle in France (EU622907) and were similar to those isolated from *H. punctata* in China (MF120940) (Figure 3A). The *B. bigemina* sequence obtain from *R. annulatus* (PZ379952) showed 99.83% identity with *B. bigemina* isolated from *R. microplus* in India (MH922762). The *B. occultans* sequence (PZ379953) exhibited 100% identity with sequences from *H. marginatum* from Tunisia (HQ331478) and Türkiye (KJ000483). The *B. caballi* sequence (PZ379948) showed 100% identity with *B. caballi* isolated from a horse in China (MH6512221) (Figure 3A).

For *Theileria*, the *T. orientalis* sequence (PZ380009) shared 100% identity with *T. orientalis* isolated from *H. longicornis* in South Korea (MT052398) and *R. microplus* in China (MH208631). The *T. ovis* sequence (PZ380010) revealed 100% identity with *T. ovis* isolated from *H. marginatum* in Türkiye (OM066225) (Figure 3B).

## 4. Discussion

This study revealed multiple *Babesia* and *Theileria* species circulating among ticks infesting animal hosts in Chuy Province, Kyrgyzstan, with an infection pattern that varied according to the tick species and host animal. The overall infection rate of 7.1% (3.0% for *Babesia* spp. and 4.0% for *Theileria* spp.) indicates considerable tick-borne parasitic pathogen pressure in this region. The infection rates were highest in hard ticks, particularly *R. annulatus* (13.8%) and *H. punctata* (12.4%). The dominance of these ticks in transmitting apicomplexan parasites has been documented in neighboring regions, such as Kazakhstan and the Xinjiang Region of China, highlighting their primary role as vectors of livestock piroplasmosis in Central Asia [22,23,24,25]. Given the importance of livestock to the national economy of Kyrgyzstan, the presence of these piroplasms in multiple tick vectors indicates a risk to veterinary health and agricultural production.

The detection of *B. caballi* in *D. marginatus*, *Dermacentor* spp., and *H. punctata* ticks is particularly noteworthy. *B. caballi* causes equine piroplasmosis, and its presence in horses restricts international horse movement and causes substantial economic loss [26]. A recent study reported a host-based *B. caballi* prevalence of 3.1% in horses in Kyrgyzstan [19]. Although tick-based and host-based prevalence estimates are not directly comparable, the results of this study similarly point to *Dermacentor* and *Haemaphysalis* species as likely vectors of this pathogen in the region. Notably, we detected *B. occultans* in a single *H. scupense* tick collected from a chicken. *B. occultans* is traditionally considered a low-pathogenicity parasite of cattle and was originally thought to be confined to the African continent but has recently been identified in the Mediterranean basin [27,28]. As this observation is based on a single positive sample, it should be interpreted with caution. *Hyalomma* frequently use birds as hosts during their immature life stages; therefore, avian species may play an underappreciated role in the geographic dispersal of *B. occultans* via migratory bird pathways [5,7]. Together with the detection of *B. bigemina* in *R. annulatus*, this unusual finding of *B. occultans* in *H. scupense* infesting a chicken demonstrates the broad ecological niche of *Babesia* species and suggests that transmission cycles in this region may extend to atypical hosts [27,29]. As this observation is based on a single positive sample, it should be interpreted with caution. The PCR detection of parasite DNA in a tick, such as in the case of *B. occultans* in *H. scupense* collected from chicken and *T. orientalis* in *Argas persicus* collected from cattle, indicates DNA carriage rather than proof of vector competence. To confirm an actual vector role for specific tick–host–pathogen combinations, further experimental transmission investigations are necessary. For example, certain detections might indicate a recent blood meal from an infected host rather than active parasite multiplication or transstadial maintenance within the tick.

*T. orientalis* was the most frequently detected pathogen in this study, carried predominantly by *R. annulatus* and *H. punctata*, and its presence in four tick genera points to a robust vector–pathogen network. This is consistent with previous surveys reporting a prevalence of *T. orientalis*, including pathogenic genotypes such as Chitose type, of up to 32.8% in cattle in Kyrgyzstan [21,30]. In contrast, *T. ovis* was detected exclusively in *H. marginatum* and *R. turanicus* collected from sheep. Although *T. ovis* is primarily regarded as a pathogen of small ruminants [31], recent detection in dogs in Kyrgyzstan highlights the potential for cross-species transmission or the involvement of atypical hosts and ticks in the epidemiological cycle [32].

The host-specific distribution of pathogens observed in this study showed that horse-derived ticks were the most frequently infected and harbored *T*. *orientalis*, *B. caballi*, and *B. bigemina*. Moreover, the detection of *T. orientalis* in *H. punctata* collected from horses, sheep and cattle suggests overlapping transmission dynamics in grazing areas where multiple livestock species coexist, highlighting the interconnected nature of tick-borne parasite transmission in this region.

This study has some limitations. First, ticks were collected in a single year (2021), which may not capture year-to-year variation in infection rates driven by climatic or seasonal factors. Second, the sample sizes varied substantially among tick species, animal hosts, and survey areas; several taxa were represented by only one or a few ticks (e.g., the single *Ornithodoros lahorensis* and the single positive sample in a chicken host), so host- and vector-specific associations involving these rare taxa should be interpreted with caution. Third, pathogen detection relied on conventional PCR targeting a limited genomic region, which may have failed to detect co-infections or low-level infections that could be detected using more sensitive quantitative PCR or metabarcoding approaches. Future studies incorporating multi-year sampling, larger and more balanced sample sizes, and higher-sensitivity molecular methods should be conducted to validate and extend these findings.

The complex interactions between ticks, animal hosts, and parasites revealed by the host–tick–pathogen and phylogenetic analyses emphasize the necessity of a One Health approach to tick-borne disease management. The *Babesia* and *Theileria* species identified in this study generally pose a low risk of human infection because no cases of human babesiosis have been reported to date. However, the diversity of *Babesia* and *Theileria* and the host–tick relationships documented in this study highlight the need for ongoing surveillance of tick-borne pathogens in both tick and vertebrate hosts. In order to develop a One Health approach for tick-borne pathogens in Kyrgyzstan, future studies should focus on the multi-host dynamics and species diversity of these parasites.

## Figures and Tables

**Table 1:** Tick-borne parasites detected in tick species infesting animal hosts from Chuy Province, Kyrgyzstan.

Host	Tick Species	No. of Ticks Sampled	No. of Positive		
			*Babesia* spp.	*Theileria* spp.	Total, *n* (%)
Cat	*Rhipicephalus turanicus*	2	0	0	0
	Subtotal	2	0	0	0
Cattle	*Argas persicus*	41	0	1	1
	*Dermacentor marginatus*	28	2	0	2
	*Dermacentor* spp.	12	2	1	3
	*Haemaphysalis punctata*	21	1	2	3
	*Hyalomma marginatum*	9	0	0	0
	*Hyalomma scupense*	2	0	0	0
	*Rhipicephalus annulatus*	9	0	0	0
	Subtotal	122	5	4	9 (7.4)
Chicken	*Argas persicus*	82	0	0	0
	*Haemaphysalis punctata*	2	0	0	0
	*Hyalomma scupense*	19	1	0	1
	*Ornithodoros lahorensis*	1	0	0	0
	Subtotal	104	1	0	1 (1.0)
Dog	*Rhipicephalus annulatus*	4	0	0	0
	*Rhipicephalus sanguineus* complex	1	0	0	0
	*Rhipicephalus turanicus*	29	0	0	0
	Subtotal	34	0	0	0
Horse	*Dermacentor* spp.	6	0	0	0
	*Haemaphysalis punctata*	20	3	2	5
	*Rhipicephalus annulatus*	45	2	6	8
	Subtotal	71	5	8	13 (18.3)
Sheep	*Argas persicus*	8	0	0	0
	*Dermacentor marginatus*	10	2	0	2
	*Dermacentor* spp.	61	1	0	1
	*Haemaphysalis punctata*	46	1	2	3
	*Hyalomma marginatum*	10	0	2	2
	*Hyalomma scupense*	1	0	0	0
	*Rhipicephalus sanguineus* complex	2	0	0	0
	*Rhipicephalus turanicus*	23	0	4	4
	Subtotal	161	4	8	12 (7.5)
Total	*Argas persicus*	131	0	1 (0.8)	1 (0.8)
	*Dermacentor marginatus*	38	4 (10.5)	0	4 (10.5)
	*Dermacentor* spp.	79	3 (3.8)	1 (1.3)	4 (5.1)
	*Haemaphysalis punctata*	89	5 (5.6)	6 (6.7)	11 (12.4)
	*Hyalomma marginatum*	19	0	2 (10.5)	2 (10.5)
	*Hyalomma scupense*	22	1 (4.5)	0	1 (4.5)
	*Ornithodoros lahorensis*	1	0	0	0
	*Rhipicephalus annulatus*	58	2 (3.4)	6 (10.3)	8 (13.8)
	*Rhipicephalus sanguineus* complex	3	0	0	0
	*Rhipicephalus turanicus*	54	0	4 (7.4)	4 (7.4)
	Total	494	15 (3.0)	20 (4.0)	35 (7.1)

## Data Availability

The newly generated sequences were submitted to the GenBank database under the accession numbers PZ379948-379953 (*Babesia* spp.) and PZ380009-PZ380010 (*Theileria* spp.). The original contributions presented in this study are included in the article. Further inquiries can be directed to the corresponding author.
